# Impact of ureido/carboxypenicillin resistance on the prognosis of ventilator-associated pneumonia due to *Pseudomonas aeruginosa*

**DOI:** 10.1186/cc10136

**Published:** 2011-04-11

**Authors:** Catherine Kaminski, Jean-François Timsit, Yohann Dubois, Jean-Ralph Zahar, Maïté Garrouste-Orgeas, Aurélien Vesin, Elie Azoulay, Céline Feger, Anne-Sylvie Dumenil, Christophe Adrie, Yves Cohen, Bernard Allaouchiche

**Affiliations:** 1 Surgical ICU, Hôpital Edouard Herriot, 5 place d'Arsonval, Lyon 69437, France; 2 Medical Polyvalent ICU, University hospital A Michallon, Bd de la Chantourne BP 217, Grenoble 39043 Cedex 9, France; 3 Integrated Research Center U823, University Grenoble 1 - Albert Bonniot Institute, Rond point de la Chantourne, La Tronche Cedex 38706, France; 4 Microbiology and Infection Control Unit, Necker Teaching Hospital, 149 rue de Sevres, Paris 75743, France; 5 Medical-Surgical ICU, Saint Joseph Hospital Network, 185 rue Raymond Losserand, Paris 75014, France; 6 Medical ICU, Saint Louis Teaching Hospital, 1 avenue Claude Vellefaux, Paris, 75010, France; 7 Outcomerea, 7 rue Louise Thuliez, Paris 75019, France; 8 Surgical ICU, Antoine Béclère Teaching Hospital, 157 rue de la porte de Trivaux, Clamart 92141, France; 9 Medical-Surgical ICU, Delafontaine Hospital, 2 rue du Dr Delafontaine, Saint-Denis 93200, France; 10 Medical-Surgical ICU, Avicenne Teaching Hospital, 125 rue de Stalingrad, Bobigny 93009, France

## Abstract

**Introduction:**

Although *Pseudomonas aeruginosa *is a leading pathogen responsible for ventilator-associated pneumonia (VAP), the excess in mortality associated with multi-resistance in patients with *P. aeruginosa *VAP (PA-VAP), taking into account confounders such as treatment adequacy and prior length of stay in the ICU, has not yet been adequately estimated.

**Methods:**

A total of 223 episodes of PA-VAP recorded into the Outcomerea database were evaluated. Patients with ureido/carboxy-resistant *P. aeruginosa *(PRPA) were compared with those with ureido/carboxy-sensitive *P. aeruginosa *(PSPA) after matching on duration of ICU stay at VAP onset and adjustment for confounders.

**Results:**

Factors associated with onset of PRPA-VAP were as follows: admission to the ICU with septic shock, broad-spectrum antimicrobials at admission, prior use of ureido/carboxypenicillin, and colonization with PRPA before infection. Adequate antimicrobial therapy was more often delayed in the PRPA group. The crude ICU mortality rate and the hospital mortality rate were not different between the PRPA and the PSPA groups. In multivariate analysis, after controlling for time in the ICU before VAP diagnosis, neither ICU death (odds ratio (OR) = 0.73; 95% confidence interval (CI): 0.32 to 1.69; *P *= 0.46) nor hospital death (OR = 0.87; 95% CI: 0.38 to 1.99; *P *= 0.74) were increased in the presence of PRPA infection. This result remained unchanged in the subgroup of 87 patients who received adequate antimicrobial treatment on the day of VAP diagnosis.

**Conclusions:**

After adjustment, and despite the more frequent delay in the initiation of an adequate antimicrobial therapy in these patients, resistance to ureido/carboxypenicillin was not associated with ICU or hospital death in patients with PA-VAP.

## Introduction

Despite many improvements in the management of mechanically-ventilated patients, ventilator-associated pneumonia (VAP) remains the second leading cause of nosocomial infections in intensive care units (ICU). VAP has one of the highest mortality rates, ranking from 20 to 50% [1], and increases length of hospital stay, and hospital costs [2].

*Pseudomonas aeruginosa *is a leading cause of nosocomial infections and one of the bacteria most frequently responsible for late-onset VAP. When VAP is documented by bronchoscopic techniques, *P. aeruginosa *is the most frequently isolated nosocomial bacteria, with more than 19% of the isolates. According to data collected by the French network REA-RAISIN during the year 2009, *P. aeruginosa *was the bacteria responsible for most nosocomial infections (17.3% of nosocomial infections) and for most VAP (22.3% of VAP) [3].

*P. aeruginosa *causes infection and damage to host tissues via the production of several extracellular virulence factors [4-7]. The *P. aeruginosa *genome is one of the largest bacterial genomes. Its large size reflects a great genetic and functional diversity, with a large number of genes predicted to encode outer membrane proteins such as those involved in adhesion, motility, antibiotic efflux and virulence factor export [8].

In addition to being intrinsically resistant to several antimicrobial agents, *P. aeruginosa *often acquires mechanisms of resistance to other antibiotics, especially in ICU patients. This increasing antibiotic resistance makes the treatment of *P. aeruginosa *ventilator-associated pneumonia (PA-VAP) more difficult and more expensive.

Few published studies report the impact of antibiotic resistance on the outcome of PA-VAP [9,10]. Their authors conclude that antibiotic resistance did not significantly affect ICU mortality. However, appropriate adjustments for differences in epidemiological and clinical characteristics between patients with resistant and susceptible infections are complex, leading to results lacking in controls for confounding factors.

The goal of this study was to estimate the mortality attributable to piperacillin resistance, while taking into account differences in the elapsed time between disease onset and the initiation of an adequate antimicrobial therapy.

## Material and methods

### Study design and data source

We conducted an exposed/non-exposed study nested in a multicenter cohort (the OUTCOMEREA^® ^database) from January 1997 to January 2008. The database, fed by 12 French ICUs, is designed to record daily disease severity and occurrence of iatrogenic events.

The senior physicians of the participating ICUs, who are closely involved in establishing the database, recorded the data daily. For each patient, the investigators entered the data into a computer case-report form using the data capture softwares VIGIREA and recently RHEA^® ^(OUTCOMEREA™, Rosny-sous-Bois, France). All records were imported to the OUTCOMEREA^® ^database. All codes and definitions were defined in writing before the start of the data collection.

The following data were collected: age, sex, comorbidities assessed according to the Acute Physiology and Chronic Health Evaluation (APACHE) II definitions [11], severity of illness both at ICU admission and daily during the ICU stay assessed using the Simplified Acute Physiology Score (SAPS) II [12] and the Logistic Organ Dysfunction (LOD) score [13], admission category (medical, scheduled surgery, or unscheduled surgery), admission diagnosis, whether the patient was transferred from a hospital ward (defined as a stay in an acute-bed ward ≥24 hours immediately before ICU admission), lengths of ICU and hospital stays, and vital status at discharge from ICU and from the hospital. Invasive procedures (placement of an arterial or central venous catheter, and endotracheal intubation), treatments of organ failure (catecholamine infusion, mechanical ventilation, hemodialysis), and antibiotic use were also captured.

Suspected VAP was defined as the development of persistent pulmonary infiltrates on chest radiographs combined with purulent tracheal secretions and/or body temperature ≥38.5°C or ≤36.5°C and/or peripheral blood leukocyte count ≥10·10^9^/L or ≤4·10^9^/L. Before receiving any new antibiotic therapy, all patients with suspected VAP underwent fiber optic bronchoscopy with a protected specimen brush and/or bronchoalveolar lavage (BAL), single-sheathed blind plugged telescopic catheter specimen collection, or tracheal aspiration, with quantitative cultures of collected specimens. The model was established using solely confirmed VAP. This was defined as a positive culture result from a protected specimen brush (≥10^3 ^cfu/ml), plugged telescopic catheter specimen (≥10^3 ^cfu/ml), BAL fluid specimen (≥10^4 ^cfu/ml), or quantitative endotracheal aspirate (≥10^5 ^cfu/ml) [14]. The investigators recorded prospectively the date of appropriate therapy start (that is, the date when at least one of the antibiotics had *in vitro *activity against the strains recovered) but the complete antimicrobial susceptibility testing results were not captured in the OUTCOMEREA ^® ^database.

A special request was performed retrospectively to collect information about antibiotic susceptibility profiles of all recovered strains of *P. aeruginosa*. Strains intermediate or resistant to one antimicrobial were considered as resistant.

The quality control processes are detailed elsewhere [14]. Briefly, it combined an initial training process, a manual, and automatic checking for inconsistencies and feedback to investigators, a data-capture training course, and a bi-annual audit of patients' files. Moreover, each ICU investigator was involved in the data analysis and study reporting.

### Study population and definitions

All patients in the database with a confirmed PA-VAP were eligible.

Patients with mechanical ventilation at admission who developed a resistant PA-VAP were compared to patients who developed sensitive PA-VAP. Among patients who contracted several episodes of PA-VAP, only the first episode was included in the analysis. We compared patients with a first episode of PA-VAP due to Ureido/carboxypenicillin sensitive (PSPA-VAP) to those having resistant strains (PRPA-VAP).

### Statistical analyses

Results are expressed as numerical values and percentages for categorical variables, and as medians and inter-quartiles (Q1 to Q3) for continuous variables.

Using an algorithm [15], we utilized a N:M matching on the duration of the ICU stay prior to PA-VAP onset.

Comparisons between matched patients were initially completed based on univariate conditional logistic regression. Multivariate conditional logistic regression was then used to examine the association between PRPA-VAP and ICU and hospital mortality. This was adjusted for potential confounding variables (that is, variables that had a *P-v*alue ≤10 in bivariate analysis). Wald χ^2 ^tests were used to determine the significance of each variable. Adjusted odds ratios (OR) and 95% confidence were calculated for each parameter estimate. A *P-*value less than .05 was considered significant. Analyses were computed using the SAS 9.1 software package (SAS Institute, Cary, NC, USA)

### Ethical issues

According to French law, this study did not require patient consent, as it involved research on a database. The study was approved by the institutional review board of the Centres d'Investigation Rhône-Alpes-Auvergne.

## Results

**Figure 1:**
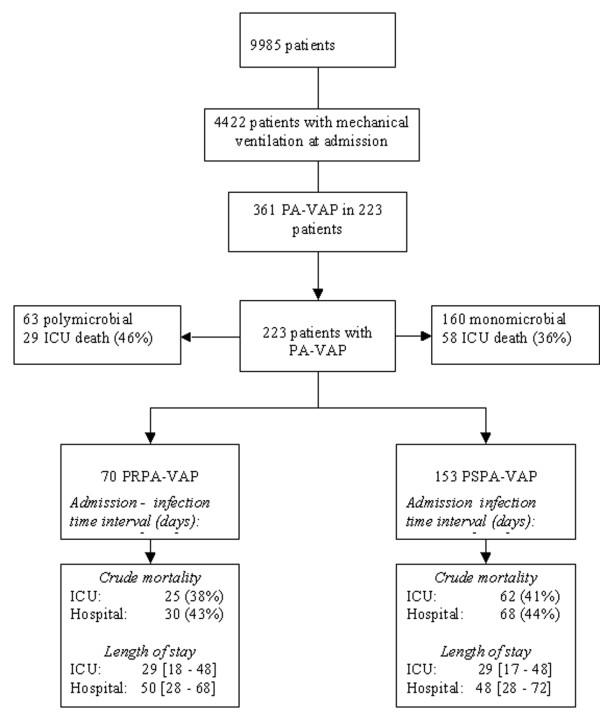
**Flowchart of the study**. PA-VAP, Ventilated Associated Pneumonia due to *Pseudomonas aeruginosa*; PRPA, piperacillin resistant *Pseudomonas aeruginosa; *PSPA, piperacillin sensitive *Pseudomonas aeruginosa*

During the study period, of the 9,985 patients included in our OUTCOMEREA ^® ^database, 4,422 received mechanical ventilation for more than two days, and 223 experienced at least one episode of PA VAP (361 episodes of PA VAP were recorded). PRPA-VAP was diagnosed in 70 patients, and PSPA-VAP was diagnosed in 153 patients (Table 1). Resistance to other antimicrobials were as follows: imipenem (25.6%, 26 not recorded), ceftazidime (83.8%, 19 not recorded), ciprofloxacin (38.5%, 55 not recorded), amikacin (17.2%, 25 not recorded), colistin (4.2%, 80 not recorded). The median length of ICU stay was 29 days. The flowchart of the study is shown in Figure 1.

**Table 1:** Risk factors of ICU death

*Variables*		*Survivors (n = 136)*	*Deaths (n = 87)*	*P*
**Male sex**		105 (77)	69 (79)	0.86
**Age**		**66 (52 to 76)**	**70 (57 to 77)**	**0.04**
**Category at admission**	**Medical**	90 (66)	62 (71)	0.49
	**Emergency surgery**	25 (18)	17 (19)	0.76
	**Scheduled surgery**	21 (15)	8 (9)	0.20
**Severity score at admission**	**LOD**	**6 (4 to 8)**	**7 (5 to 10)**	**0.04**
	**SAPS II**	**45 (36 to 53)**	**54 (42 to 66)**	**0.0001**
	**SOFA**	**8 (6 to 10)**	**9 (6 to 11)**	**0.03**
**Duration of ICU stay before VAP**		10 (6 to 18)	9 (5 to 18)	0.13
**Duration of stay**	**ICU**	32 (20 to 50)	26(14 to 41)	0.19
	**Hospital**	**57 (42 to 84)**	**30 (19 to 59)**	**<0.0001**
**Main diagnosis at admission**	**Septic shock**	25 (18)	18 (21)	0.74
	**Multiple organ failure**	6 (4)	6 (7)	0.26
	**Cardiac failure**	5 (4)	2 (2)	0.53
	**Acute respiratory failure**	47 (35)	27 (31)	0.59
	**COPD exacerbation**	9 (7)	5 (6)	0.96
	**Acute renal failure**	3 (2)	2 (2)	0.90
	**Scheduled surgery**	9 (7)	1 (1)	0.08
**Chronic illness**	**Cardiovascular**	**16 (12)**	**19 (22)**	**0.03**
	**Pulmonary**	31 (23)	22 (25)	0.58
	**Renal**	6 (4)	4 (5)	0.90
	**Hepatic**	6 (4)	7 (8)	0.29
	**Diabetes**	20 (15)	9 (10)	0.73
	**At least one chronic illness**	**59 (43)**	**51 (59)**	**0.02**
**Treatment at admission**	**Vasopressors**	84 (62)	59 (68)	0.30
	**Steroids**	39 (29)	35 (40)	0.05
	**Broad-spectrum antimicrobials**	88 (65)	58 (67)	0.45
	**Hemodialysis/hemofiltration**	14 (10)	8 (9)	0.91
**Polymicrobial VAP**		34 (25)	29 (33)	0.27
**SOFA score at VAP onset**		5 (3 to 7)	7 (4 to 9)	0,0004
Adequate antimicrobial treatment*	**J 0**	53 (39)	34 (39)	0.42
	**J+1**	41 (30)	25 (29)	0.66
	**J+2**	32 (23)	19 (22)	0.40
**Antimicrobials**	**Monotherapy**	15 (12)	17 (22)	0.12
	**Bi or Tri antibiotic therapy**	111 (82)	61 (70)	0.11

Data are the number (%) of patients (minimum to maximum). ICU, intensive care unit; LOD, logistic organ dysfunction; PRPA, piperacillin resistant *Pseudomonas aeruginosa*; PSPA, piperacillin sensitive *Pseudomonas aeruginosa*; SAPS II, Simplified Acute Physiology Score version II; SOFA, Simplified Organ Failure Assessment; VAP, ventilator-associated pneumonia.

(*) nb of calendar day between the suspicion of VAP/bacteriological sampling and the initiation of an antimicrobial treatment effective on recovered micro-organisms

### Factors associated with Ureido-/carboxypenicillin resistance

Clinical characteristics at ICU admission and within 48 hours before VAP diagnosis for PRPA and PSPA-VAP are listed in Table 2. The groups were similar with regard to sex, age, SAPS II, immunosuppression, underlying diseases and proportions of medical and surgical patients.

**Table 2:** Characteristics of patients with *Pseudomonas aeruginosa *ventilator-associated pneumonia at admission to the intensive care unit

*Characteristics*		*PSPA-VAP N = 153*	*PRPA-VAP N = 70*	***P***^**a**^
**Male sex**		119 (78)	55 (79)	0.75
**Age**		68 (55 to 76)	66 (49 to 77)	0.54
**Type of admission**	**Medical**	106 (69)	46 (66)	0.83
	**Surgical**	27 (18)	15 (21)	0.53
	**Scheduled**	20 (13)	9 (13)	0.65
**SAPS II**		48 (38 to 61)	48 (38 to 55)	0.80
**Main diagnosis at admission**	**Septic shock**	**23 (15)**	**20 (29)**	**0.02**
	**Multiple organ failure**	8 (5)	4 (6)	0.79
	**Cardiovascular failure**	6 (4)	1 (1)	0.64
	**Respiratory failure**	55 (36)	19 (27)	0.14
	**Acute respiratory exacerbation of chronic pulmonary diseases**	9 (6)	5 (7)	0.61
	**Acute renal failure**	4 (3)	1 (1)	0.57
	**Scheduled surgery**	**4 (3)**	**6 (9)**	**0.02**
**Treatments within 48 hrs of ICU admission**	**Vasopressors**	101 (66)	42 (60)	0.25
	**Steroids**	50 (33)	24 (34)	0.67
	**Broad-spectrum antimicrobials**	**92 (60)**	**54 (77)**	**0.03**
**Duration of ICU stay before VAP**		9 (6 to 17)	11 (6 to 18)	0.23
**Organ dysfunction scores the day before VAP**	LOD	5 (3 to 7)	4.5 (3 to 7)	0.56
	SOFA	6 (3 to 8)	5 (3 to 7)	0.77
**Polymicrobial VAP**		45 (29)	18 (26)	0.53
**Positive blood culture**		6 (4)	7 (10)	0.11
**Duration of stay**	**ICU**	29 (17 to 48)	28,5 (18 to 48)	0.37
	**Hospital**	47.5 (28 to 72)	50 (28 to 68)	0.24

Data are the number (%) of patients (minimum to maximum). ICU, intensive care unit; LOD, logistic organ dysfunction; PRPA, piperacillin resistant *Pseudomonas aeruginosa*; PSPA, piperacillin sensitive *Pseudomonas aeruginosa*; SAPS II, Simplified Acute Physiology Score version II; SOFA, Simplified Organ Failure Assessment; VAP, ventilator-associated pneumonia.

^a ^Wald χ^2 ^test in conditional regression

Patients with PRPA-VAP were more likely to have septic shock at ICU admission (28.6% (20 of 70 patients) vs. 15% (23 of 153 patients); *P *= 0.02), and to have a previous carriage or colonization with a multiresistant strain of PA. Positive blood culture (between Day -2 VAP diagnosis and Day +2) was more frequent in the PRPA-VAP than in the PSPA-VAP group (10% (7 of 70 patients) vs. 3.9% (6 of 153 patients); *P *= 0.054).

Details of antimicrobials received prior to VAP infection are listed in Table 3. Compared to patients with PSPA-VAP, patients with PRPA-VAP were significantly more likely to have received broad-spectrum antimicrobials during their admission in the ICU (77.1% (54 of 70 patients) vs. 60.1% (92 of 153 patients); *P *= 0.03). Before VAP diagnosis, patients with PRPA-VAP were more likely to have received tazobactam therapy (18.6% (13 of 70 patients) vs. 9.2% (14 of 153 patients); *P *= 0.01), and to have received ureidopenicillins or carboxypenicillins therapy (31.4% (22 of 70 patients) vs. 13.1% (20 of 153 patients); *P *= 0.0004). Differences in the use of fluoroquinolones therapy did not reach statistical significance (24.3% in PRPA group (7 of 70 patients) vs. 13.1% in PSPA group (20 of 153 patients); *P *= 0.058).

**Table 3:** Antimicrobials received in the ICU within the seven days prior to VAP onset

Antimicrobials	PSPA-VAP (*n *= 153)	PRPA-VAP (*n *= 70)	*P*-value
Penicillin	63 (41)	21 (30)	0.27
Cephalosporins 1, 2 or 3	46 (30)	22 (31)	0.99
Ceftazidime	7 (5)	3 (4)	0.99
Cephalosporins 4 (cefepim, cefpirome)	4 (3)	5 (7)	0.19
Piperacillin-Tazobactam	14 (9)	13 (19)	0.01
Ureidopenicillin - carboxypenicillin	20 (13)	22 (31)	0.0004
Tazobactam, sulbactam or Clavulanic acid	56 (37)	26 (37.1)	0.41
Penems	11 (7)	6 (8.6)	0.92
Fluoroquinolones	20 (13)	17 (24.3)	0.058
Aminoglycosides	38 (25)	21 (30)	0.24
Azoles	16 (10)	13 (19)	0.14
Glycopeptides	24 (16)	14 (20)	0.42
Other	14 (9)	8 (11)	0.99
At least one antimicrobial	101 (66)	44 (63)	0.60

Data are the numbers (%).

Percentage of antibiotic-free days was different between the two groups for tazobactam, and ureidopenicillins-carboxypenicillins. Compared with patients with PRPA-VAP, patients with PSPA-VAP had more tazobactam-free days (*P *= 0.019), and less ureidopenicillins-carboxypenicillins-free days (*P *= 0.007).

Adequate antibiotic therapy was started within 24 h after the diagnosis of VAP for 36 patients (51.4%) in the PRPA-VAP group, versus 117 patients (76.5%) in the PSPA-VAP group (*P *= 0.001). Adequate antibiotic therapy was started at least two days after PA VAP diagnosis for 25 patients (35.7%) in the PRPA-VAP group, versus for 26 patients (17%) in the PSPA-VAP group (*P *= 0.007). Use of bi- or tri-antimicrobial-therapy was similar between groups. Antibiotic therapy before ICU discharge was not adequate for 9 patients (12.9%) in the PRPA-VAP group, versus 10 patients (6.5%) in the PSPA-VAP group (*P *= 0.054).

The rate of recurrence was not influenced by resistance (PSPA-VAP 28 (18.3%) vs PRPA-VAP 11 (15.7%), *P *= 0.83).

Compared with patients with PSPA-VAP, PRPA-VAP patients had similar lengths of ICU stay prior to VAP (11 days (range, 6 to 18 days) vs. 9 days (range, 6 to 17 days); *P *= 0.53). PRPA-VAP and PSPA-VAP were associated with similar crude ICU mortality (38% (25 of 70 patients) vs. 41% (62 of 153 patients); *P *= 0.56) as well as in hospital mortality (43% (30 of 70 patients) vs. 44% (68 of 153 patients); *P *= 0.85).

### Risk factors for death

Risk factors for ICU death are listed in Table 1. Risk factors found for ICU death at admission were: age, at least one chronic illness, admission for cardiac illness or septic shock, Simplified Acute Physiology Score version II (SAPS II), organ dysfunction scores (LOD, SOFA). Two days before PA-VAP, risk factors for ICU death were: treatment with vasopressors, treatment with steroids, SAPS II, and organ dysfunction scores (LOD, SOFA).

Resistance to Ureido/carboxypenicillin was not a risk factor for ICU death. After adjusting for other risk factors of death, differences between groups and duration of ICU stay prior to the VAP onset, resistance to ureido/carboxypenicillins was no more a risk factor for death (Table 4).

**Table 4:** Multivariate analysis, factors associated with ICU and hospital death after adjusting on potential confounding factors

	OR	95% CI	*P*-value
*ICU death*			
At least one chronic illness	2.47	1.24 to 4.92	0.01
Fluoroquinolones prior VAP onset	2.45	0.91 to 6.63	0.08
Positive blood culture	5.11	1.16 to 22.6	0.03
LOD two days before infection (per point)	1.17	1.01 to 1.37	0.04
SAPS at admission (per point)	1.03	1.00 to 1.05	0.04
*PRPA-VAP*	*0.73*	*0.32 to 1.69*	*0.46*
*Hospital Death*			
At least one chronic illness	2.29	1.16 to 4.54	0.02
Fluoroquinolones prior VAP onset	2.89	1.04 to 8.04	0.04
Positive blood culture	4.58	0.96 to 21.9	0.05
SAPS II at admission	1.04	1.01 to 1.06	0.01
*PRPA-VAP*	*0.87*	*0.38 to 1.99*	*0.74*

**Figure 2:**
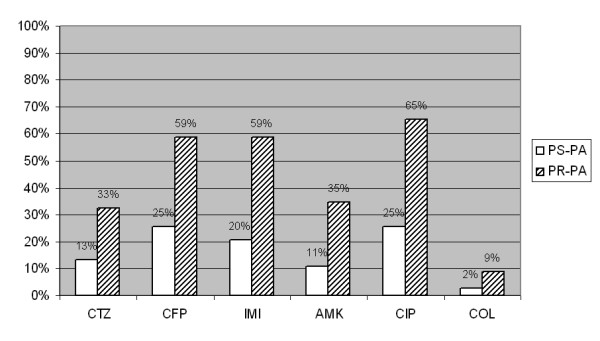
**Resistance to other antimicrobials of ureido/carboxy susceptible and resistant strains (*n *= 129)**. AMK, amikacine; CFP, cefepime; CIP, ciprofloxacin; COL, colimycin; CTZ, Ceftazidime; IMI, Imipenem; PR-PA piperacillin-resistant *P. aeruginosa*; PSPA, piperacillin-susceptible *P. aeruginosa*; (*) Missing values: some antibiotics were not tested by the microbiology lab or not found, CTZ 2 (1%), CFP 63 (31%), IMI 7 (3%), CIP 10 (5%), AMK 8 (4%), COL 60 (30%). All the differences were statistically significant (*P *< 0.05).

Similarly, resistance to imipenem, ceftazidime, amikacin, ciprofloxacin and colistin (Figure 2) was not associated with ICU death or hospital death.

The results remained unchanged when analysis was restricted to the 87 patients adequately treated the day of the infection onset (OR for ICU mortality 1.22 (95% CI, 0.31 to 4.78; *P *= 0.78); OR for hospital mortality, 1.10 (95% CI, 0.29 to 4.10; *P *= 0.89)).

## Discussion

To date, our study is one of the largest to have evaluated the impact of piperacillin resistance in PA-VAP [9,10]. All data were carefully recorded by senior physicians on computer forms. Definitions and antimicrobial treatments were in accordance with international guidelines. The major result is that piperacillin resistance is associated with a higher rate of inappropriate antimicrobial therapy. Unadjusted mortality was similar in PSPA-VAP and PRPA-VAP groups. After careful adjustment for time in the ICU at VAP diagnosis and for parameters that differ between the PRPA and the PSPA groups (severity at admission, previous antibiotic treatment, and adequacy of antimicrobial treatment), piperacillin resistance was found to not be associated with ICU or hospital death in the multivariate logistic regression analysis.

We observed a high rate of *P. aeruginosa *strains resistant to piperacillin (31%). This is in agreement with previous studies conducted in Europe [16]. The percentage of *P. aeruginosa *resistance to ureidopenicillin reached 37% in the EPIC I study which included only bacterial strains from the European ICU [17]. The pathogenicity of *P. aeruginosa *is multifactorial, strain-specific, and dependent on complex host factors. Morbidity and mortality for patients infected with piperacillin resistant *P. aeruginosa *might be related to the virulence of the bacteria but also to the antimicrobial treatment administered. Both virulence factor genes and antimicrobial resistance genes are mostly carried by transposons and integrons, that is, genetic entities able to mediate their own translocation from one DNA site to another one. Integrons are particularly dominant contributors to the development of multi-drug resistant *P. aeruginosa *strains [8].

Conceivably PRPA strains may be more virulent than PSPA strains. *P. aeruginosa *exhibits multiple virulence factors: some surface factors including flagellum, pili, LPS, some secreted factors including a type III secretion system that confers the ability to inject toxins into host cells; quorum-sensing molecules (a complex regulatory circuit involving cell-to-cell signalling) and alginate. Some studies conducted *in vitro *and in experimental models found that virulence of *P. aeruginosa *might be reduced in mutant resistant strains of *P. aeruginosa *suggesting that antibiotic resistance imposes a fitness cost on the bacteria [18,19]. Recent data on *in vitro *mutants have suggested that virulence of *P. aeruginosa *might be reduced when *mex *efflux systems are overexpressed [20]. Indeed, it seems that mutant strains may recover their fitness or virulence by compensatory mutations. Hoquet *et al. *conducted a study on 120 strains of *P. aeruginosa *from episodes of septicaemia: 75% of strains displayed a significant resistance to one or more of the tested antimicrobials. *P. aeruginosa *may accumulate intrinsic (chromosomal) and exogenous resistance mechanisms without losing its ability to generate severe bloodstream infections [21].

Jeannot *et al. *focused on clinical isolates of *P. aeruginosa *mexCD-oprJ overproducing efflux mutants: mexCD-oprJ up regulation (which correlated with increase resistance to ciprofloxacin and cefepime, increased susceptibility to ticarcillin, aztreonam, imipenem and aminoglycosides), associated with impaired bacterial fitness although it was isolated from confirmed cases of clinical infections [22].

In our study, 153 of 223 patients received adequate antibiotic therapy within 24 hours after pneumonia suspicion (51.4% in the PRPA group and 76.5% in the PSPA group). Garnacho-Montero *et al. *[23] evaluated the impact on the outcome of a monotherapy or a combined therapy in patients with PA-VAP. They showed that the initial use of a combination therapy significantly reduced the likelihood of inappropriate therapy, which was associated with a higher risk of death. However, the administration of only one effective antimicrobial or combination therapy provided similar outcomes. For Kang *et al. *adequate initial antibiotic therapy appeared to be one of the most important factors in the treatment of severe *P. aeruginosa *infections, as they observed a trend towards higher mortality rates as the interval prior to appropriate treatment increased. However, their results were not statistically significant [24].

In our study, antibiotic resistant strains were associated with an increased risk of inadequate antimicrobial therapy, but we did not find that PRPA influenced recurrence, relapse or mortality.

We previously showed that the highest impact of inappropriate therapy on prognosis obtained when patients' severity score is intermediate [25,26]. The absence of impact of inappropriate antimicrobial therapy on the prognosis might be due to the high severity of patients at VAP onset (SOFA at 6 in median in the present study).

As any delay in the initiation of an adequate antimicrobial therapy is known to be a major prognostic risk factor in nosocomial infection due to *P. aeruginosa *[24,27], the absence of an increased mortality, where the initial antibiotic therapy is less often appropriate, would plead in favour of impaired virulence of the resistant strains.

In order to control this potential confounding factor, we analysed the subgroup of 87 patients adequately treated within 24 hours after VAP diagnosis. In the multivariate analysis, the only risk factor of ICU or hospital death was having at least one chronic illness, not piperacillin resistance.

Other studies comparing outcomes of susceptible and resistant *P. aeruginosa *infections are scarce. Recently, Combes *et al. *conducted a *post-hoc *analysis of the PNEUMA randomized study, which involved only cases of adequately treated PA VAP [9]. PRPA-VAP was associated with a higher mortality than PSPA-VAP. However, after adjustment for age, female gender, underlying comorbidities, and SOFA score, piperacillin resistance was no longer associated with mortality (OR, 2.00; 95% CI, 0.72 to 5.61; *P *= 0.194). Similarly, in a retrospective cohort study evaluating the epidemiological characteristics of 135 episodes of VAP caused by PRPA or PSPA, Trouillet *et al. *did not find any increased death rates for PRPA infections [10]. Data from the few studies concerning *P.aeruginosa *bacteraemia reported similar results. Blot *et al. *conducted a retrospective study on antibiotic-susceptible and antibiotic-resistant nosocomial gram-negative bacilli bacteraemia in critically ill patients. Factors associated with in-hospital mortality were the age, a high-risk source of bacteraemia, and APACHE II score, but not antibiotic resistance [28]. This result is in accordance with other studies conducted about gram-negative bacilli infection [29], as well as *Staphylococcus aureus *pneumonia [30].

Some studies have described higher mortality rates in association with infections caused by antibiotic resistant *P. aeruginosa*. In a retrospective matched control study by Aloush *et al.*, patients infected with multi-resistant *P. aeruginosa *strains had higher mortality rates and increased durations of hospital stay, but the control patients did not have *P. aeruginosa *infections [31]. In an older study evaluating health and economic outcomes of resistant *Pseudomonas *infections, the same group found that, although the patients with resistant strain at hospital admission did not have a poorer overall prognosis, the emergence of resistant strains during the hospital stay was associated with a prolonged length of stay and a higher hospital mortality rate [32].

Our study highlighted the major role of previous antibiotic therapy. The emergence of PRPA: PRPA VAP episodes were significantly associated with the administration of broad-spectrum antimicrobials at admission to ICU, such as ureidopenicillins, carboxypenicillins or fluoroquinolones. These findings are in accordance with the results of Harris *et al. *[33], who found that the piperacillin-tazobactam exposure was the major factor that predispose for the development of an infection with a multidrug resistant *P. aeruginosa*. In a clinical study, Reinhardt *et al. *found that resistant *P. aeruginosa *is selected in less than seven days in patients treated with piperacillin-tazobactam [34].

In contrast to previous studies [32,33,35], in our work, imipenem was not associated with VAP due to PRPA. But the main resistance mechanism to carbapenem is loss of the porin oprD, which leads to a selective resistance to these antibiotics.

Our study confirms results of the case-control study conducted by Harris *et al. *in that previous exposure to piperacillin: piperacillin-tazobactam was statistically associated with the isolation of piperacillin-tazobactam-resistant *P. aeruginosa *(OR 8.63; 95% CI 6.11 to 12.20; *P *< 0.0001) [33]. In an observational study comparing the relative risks of emergence of resistant *P. aeruginosa *associated with four individual antipseudomonal agents, Carmeli *et al. *demonstrated that there was an significant association between piperacillin treatment and the emergence of piperacillin resistance (HR = 5.2; *P *= 0.01) [32].

In our study, fluoroquinolones treatment in the week prior to VAP was an independent factor associated with hospital death. Few data could explain this result. The expression of mexAB-oprM, responsible for acquired resistance to fluoroquinolones is regulated by the quorum-sensing, and, therefore, is known to be growth-phase dependent [36,37]. We suggest that the overexpression of the porin mexAB-oprM could be synchronized with a hyper-production of bacterial virulence factors, leading to a very virulent bacterial inoculum.

Results on the role of fluoroquinolones in the emergence of piperacillin-resistant *P. aeruginosa *were of borderline statistical significance. Trouillet *et al. *suggested that receiving any fluoroquinolone may be a risk factor for acquiring piperacillin-resistant *P. aeruginosa *[10]. In a cohort study, Carmeli *et al. *also found previous ciprofloxacin treatment was a risk factor of the emergence of antibiotic-resistant *P. aeruginosa *(hazard ratio 9.2; *P *= 0.04) [32]. But in contrast to these previous studies, we did not found any role of carpapenems in the emergence of PRPA.

Duration of exposure to these antibiotics should also be taken into consideration. In a case-control study conducted by Paramythiotou *et al.*, among 34 patients with multi-drug resistant *P. aeruginosa*, a previous treatment with ciprofloxacin or imipenem was a significant risk factor for the acquisition of multi-drug resistance only when the duration of the treatment was longer than the median duration of treatment with these antimicrobials observed in that study [38].

Our study has several limitations. First, we used data prospectively collected in a large database that was not specifically designed for the present subject. However, all data were collected prospectively, with special attention to nosocomial infections and treatment adequacy. VAP was consistently documented by quantitative cultures of distal pulmonary specimens, and all patients were observed until ICU and hospital discharge.

Second, we have no data about MICs of strains to antimicrobials and we did not determine plasma antimicrobial levels. PK/PD optimization may have affected the outcome of VAP. However, antimicrobials used in the study's ICUs comply with international guidelines and aminoglycosides were used when appropriate.

## Conclusions

In summary, after controlling for the duration of mechanical ventilation before VAP, piperacillin resistance is not significantly associated with ICU death or hospital death in patients with PA-VAP, despite the more frequent delay in the initiation of an adequate antimicrobial therapy observed in the PRPA group. This result pleads in favour of an impaired virulence of the resistant strains of *P. aeruginosa*, and should be confirmed by further studies conducted by physicians and bacteriologists.

## Key messages

• *P. aeruginosa *resistance to ureido/carboxypenicillin is associated with a significant decrease of adequacy of probabilistic antimicrobial therapy.

• The absence of over-mortality associated with resistance may suggest a lower virulence of the more resistant pseudomonas aeruginosa strains.

## Abbreviations

APACHE: Acute Physiology and Chronic Health Evaluation; BAL: bronchoalveolar lavage; LOD: logistic organ dysfunction score; PA-VAP: *Pseudomonas aeruginosa *ventilator-associated pneumonia; PRPA: ureido/carboxy resistant *Pseudomonas aeruginosa*; PSPA: ureido/carboxy sensitive *Pseudomonas aeruginosa*; SAPS: Simplified Acute Physiology Score; SOFA: Sequential Organ Failure Assessment; VAP: ventilator-associated pneumonia.

## Competing interests

The authors declare that they have no competing interests.

## Authors' contributions

CK participated in the study design and in the redaction of the draft and the final manuscript. JFT conceived the study design and coordinated the data-capture, the data cleaning, the statistical analysis and the redaction of the final manuscript. YD, JRZ, MGO, EA, ASD, CA and YC participated in the patients enrolment into the study. CF participated in study design, and in the redaction of the final manuscript. AV performed the statistical analysis. BA coordinated the redaction of the final manuscript. All the authors read and approved the final manuscript.
